# Prediction of stress-strain behavior of rock materials under biaxial compression using a deep learning approach

**DOI:** 10.1371/journal.pone.0321478

**Published:** 2025-04-29

**Authors:** Changsheng Li, Xinsong Zhang

**Affiliations:** 1 State Key Laboratory of Shale Oil and Gas Enrichment Mechanisms and Efficient Development, Beijing, China,; 2 SINOPEC Key Laboratory of Geology and Resources in Deep Stratum, Beijing, China,; 3 School of Earth Sciences, East China University of Technology, Nanchang, China; Universiti Teknologi Petronas: Universiti Teknologi PETRONAS, MALAYSIA

## Abstract

Deep learning has significantly advanced in predicting stress-strain curves. However, due to the complex mechanical properties of rock materials, existing deep learning methods have the problem of insufficient accuracy in predicting the stress-strain curves of rock materials. This paper proposes a deep learning method based on a long short-term memory autoencoder (LSTM-AE) for predicting stress-strain curves of rock materials in discrete element numerical simulations. The LSTM-AE approach uses the LSTM network to construct both the encoder and decoder, where the encoder extracts features from the input data and the decoder generates the target sequence for prediction. The mean square error (*MSE*), root mean square error (*RMSE*), mean absolute error (*MAE*), and coefficient of determination (*R*^2^) of the predicted and true values are used as the evaluation metrics. The proposed LSTM-AE network is compared with the LSTM network, recurrent neural network (RNN), BP neural network (BPNN), and XGBoost model. The results indicate that the accuracy of the proposed LSTM-AE network outperforms LSTM, RNN, BPNN, and XGBoost. Furthermore, the robustness of the LSTM-AE network is confirmed by predicting 10 sets of special samples. However, the scalability of the LSTM-AE network in handling large datasets and its applicability to predicting laboratory datasets need further verification. Nevertheless, this study provides a valuable reference for solving the prediction accuracy of stress-strain curves in rock materials.

## 1. Introduction

Stress-strain curves can reveal the mechanical behavior of materials under external forces. Analyzing the stress-strain behavior of rock materials is crucial for evaluating safety in geotechnical engineering, e.g., stability analysis of rock slopes [1,2] and sensitivity studies of geological hazards [3,4]. Currently, the discrete element method (DEM) is widely used to model the stress-strain behavior of rock materials [5–8]. However, over the past decades, constitutive models based on mechanical assumptions have often been used to capture the mechanical behavior of rock materials [9]. Due to the complex mechanical characteristics of rock materials, the development of a unified theoretical model remains an ongoing challenge [10–14]. Particularly, most constitutive models typically require many parameters that are sometimes difficult to determine [15–17].

With the advancement of machine learning, obtaining constitutive relationships for materials through data-driven methods is a potential scheme to address the above challenge [18–21]. Compared to traditional laboratory and numerical simulation methods, data-driven deep learning approaches can quickly predict the nonlinear behavior of materials under different conditions. Deep learning methods leverage multi-layer neural network structures that can effectively address highly complex nonlinear problems through automated feature learning and end-to-end training. These methods excel in capturing nonlinear relationships. However, they come with higher computational costs and longer training times. In contrast, traditional machine learning methods offer better computational efficiency and easier implementation, making them widely applicable across various fields such as granular materials [22], energy storage [23], computational physiology [24], and renewable energy [25]. However, they tend to be less accurate when handling large-scale datasets or complex problems [26]. Therefore, deep learning methods are particularly suited for analyzing stress-strain data of different materials with strong nonlinear characteristics. Ghaboussi et al. [27] modeled the mechanical behavior of concrete using neural networks to predict various load paths under biaxial loading. Ellis et al. [28] simulated the stress-strain characteristics of soil using neural networks. Banimahd et al. [29] used artificial neural networks to model the stress-strain behavior of in situ sandy soils containing non-plastic fines and pointed out the problem that the ANN model does not provide information on how the inputs affect the outputs. However, the constitutive behavior fundamentally involves a time sequence problem [30]. Considering the historical behavior of stress-strain enhances prediction accuracy. Significant progress in artificial intelligence has been made for time series forecasting problems [31–33]. Models such as recurrent neural networks (RNNs) and the long short-term memory neural network (LSTM) can effectively handle long-term dependencies and yield favorable results in time series forecasting [34–35]. So far, temporal neural networks have been widely used to capture the mechanical behavior of various materials. Zhang et al. [17] employed the LSTM model to reproduce the stress-strain behavior of soil, discovering a novel phenomenon of “bias at low-stress levels” when modeling the stress-strain behavior of soil using LSTM, namely, the bias in the predicted results is greater at low-stress levels. Wang et al. [18] explored and validated the capabilities of recurrent neural networks in predicting the constitutive behavior of granular materials by constructing a temporal convolutional network. Shi et al. [36] predicted the deformation of rock materials under different loading conditions using LSTM, proposing a method to integrate the trained LSTM model as a constitutive relationship within finite element methods (FEM) to model the mechanical behavior of sandstone. Li et al. [37] proposed a fractional long short-term memory (F-LSTM) neural network prediction model, which effectively addresses the lengthy process of obtaining stress-strain behavior during the stretching of rubber. Li et al. [38] proposed a data-driven method based on the LSTM model to simulate the mechanical response of frozen soil.

Although the LSTM method has been widely used to capture the mechanical behavior of various materials, it has rarely been applied to the study of large-scale rock mass mechanical properties. Additionally, existing studies have shown a significant bias in the prediction results of traditional neural network models and LSTM models at low-stress levels. This problem is relevant with the error back propagation in the training process, where the small weight gradients of the sample at low-stress levels can lead to insufficient learning of these samples, resulting in large prediction errors [17]. Consequently, the accuracy of stress-strain curve prediction remains a problem waiting to be solved. To address this issue, this paper introduces a novel LSTM Autoencoder (LSTM-AE) model for predicting the stress-strain behavior of rock materials in DEM numerical simulations. Unlike traditional LSTM models, the LSTM-AE model improves prediction accuracy by incorporating an autoencoder structure and utilizing LSTM units to construct the encoder and decoder. The following are the main contributions of this research work:

1. An LSTM-AE network is proposed in this paper for predicting the stress-strain curve of rock materials in DEM numerical simulations. In the proposed model, the encoder and decoder are implemented using LSTM cells, which effectively capture temporal dependencies between sequences.
2. The proposed LSTM-AE network is compared with LSTM, RNN, BPNN, and XGBoost models to analyze their prediction accuracy and validate the effectiveness of the proposed model.
3. The robustness of the proposed model is analyzed, and the impact of microscopic parameters in DEM on the model’s prediction accuracy is explored, thereby verifying its reliability in predicting special samples.

## 2. Discrete element modeling and data preparation

### 2.1. Discrete element modeling of sample

**Fig 1:**
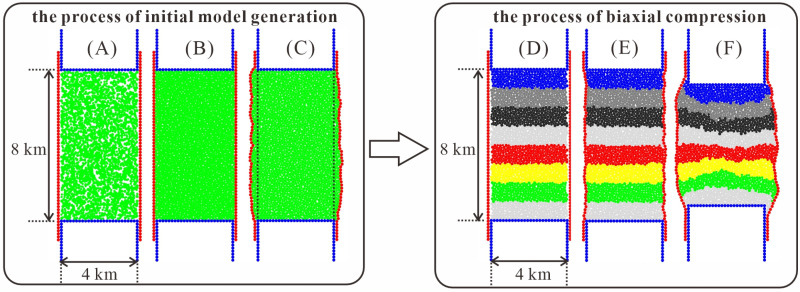
The process of discrete element modeling [ 42,47,48].

In this paper, the Hertz-Mindlin contact mechanics model and the bond contact mechanics model are employed to simulate the deformation behavior of brittle rocks. The combination of the Hertz-Mindlin model with bond contact effectively accounts for the bonding characteristics between particles, thereby accurately simulating the formation and expansion of rock cracks. Compared with other models, it can better reflect the mechanical properties of rock materials and is more suitable for simulating rock mass failure [39]. The dataset used for model training and testing was generated through discrete element numerical simulations. Biaxial compression tests were conducted using the high-performance 2D discrete element software ZDEM [40–44], which produced stress-strain curve data. However, at large scales, rock mass often exhibits fractures and cracks [45]. Therefore, selecting appropriate particle properties in the DEM is crucial to reproducing the actual mechanical behavior of the rock mass [46]. In order to simulate the structural deformation behavior of geological layers in nature and accurately characterize the mechanical properties of the rock mass at this scale, biaxial specimens measuring 4 km × 8 km were constructed in the expectation of simulating the overall mechanical properties of the rock mass [42,46,47]. The microscopic parameters used in the biaxial test are referenced in Li [42] and Morgan [47]. Modeling is divided into two main processes, the process of initial model generation and the process of biaxial compression (Fig 1).

The initial model generation of biaxial specimens employed the radii expansion method [42,49,50]. The process is as follows: First, 2100 particles with radii of 30 and 40 meters are randomly generated in a 4×8 km area, where the number of particles of both types is approximately the same, as shown in Fig 1A. Next, the microscopic parameters of the rock material were assigned to the particles, and the values of the microscopic parameters are shown in Table 1. The particle radii are then doubled using the radii expansion method, and a system in equilibrium was obtained by calculation as shown in Fig 1B. Finally, the walls on both sides are loosened, allowing the system to expand again under a given confining pressure (*σ*_*3*_). The model in the dashed line is derived as the initial model, as shown in Fig 1C.

**Table 1:** Microscopic parameters of particles [42,47].

Parameters	Value
*d (*m)	120,160
*G (*Pa)	2.9e^9^
*ρ(*kg∙m^-3^)	2.5e^3^
*g (*m∙s^-2^)	9.8
υ	0.2
*dt (*s)	5.0e^-2^
*α*	0.4
*μ*	0 ~ 3.0

*Note: d, largest particle diameter. G, shear modulus of particles. *ρ*, particle density. g, gravitational acceleration. *υ*, poisson’s ratio. dt, time setp. α, local daming coefficient. μ, friction coefficient.*

The biaxial compression process is described as follows: The initial model was placed into an area of the same size, and the microscopic parameters of the particles were set as shown in Table 1. In addition, the microscopic and bond parameters of the particles influence the overall mechanical properties of the specimen. Therefore, the bond parameters [42,47] of the rock layers were set as shown in Table 2. Subsequently, biaxial compression tests were conducted, and the deformation of the rock material was recorded. Finally, stress-strain data were collected for a series of biaxial compression tests.

**Table 2:** The bond parameters for rock layers particles [42,47].

Parameters	Value
*E*_*b*_(Pa)	2.0e^8^
*G*_*b*_(Pa)	2.0e^8^
*T*_*b*_(Pa)	0 ~ 4.0e^7^
*C*_*b*_(Pa)	0 ~ 8.0e^7^

*Note: E*_*b*_ *, Young’s modulus. G*_*b*_ *, shear modulus. T*_*b*_ *, tensile strength. C*_*b*_ *, shear strength.*

### 2.2. Data preparation

**Fig 2:**
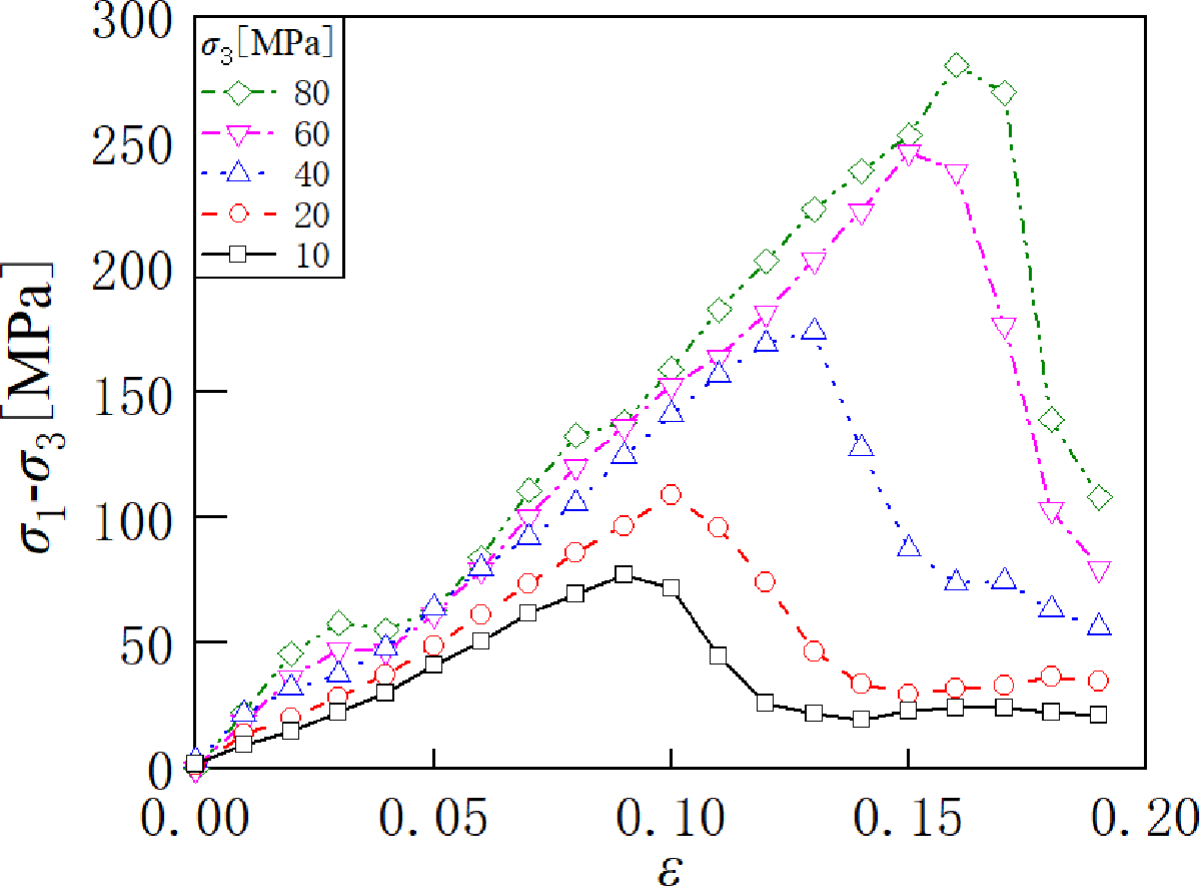
The stress-strain curves obtained by biaxial compression test.

Discrete element simulations were conducted through the above modeling process. However, different microscopic parameters affect the mechanical properties of rock material in various ways [51]. To acquire more stress-strain data, 96 sets of biaxial compression tests were randomly carried out at five confining pressures with different friction coefficients, different tensile strengths, and different shear strengths, respectively. Each sample contains 20 loading steps, which means 20 stress-strain pairs. Therefore, this means that there are 9600 (96×5×20) pairs of stress-strain pairs as data. Fig 2 illustrates the stress-strain curves obtained from biaxial compression tests at five different confining pressures.

## 3. Methodology

### 3.1. Long short-term memory neural networks

**Fig 3:**
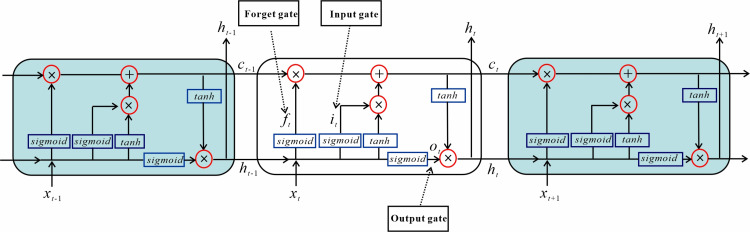
The structure of LSTM network [ 61].

In recent years, deep learning has achieved significant advancements in processing nonlinear and high-dimensional data, owing to its data processing capabilities and automatic feature extraction advantages [52]. By constructing multi-layer neural networks, deep learning can autonomously extract higher-order features from data and recognize complex patterns. This ability makes it perform well in complex tasks [53–56]. However, in time series analysis, a key challenge for deep learning is effectively capturing long-term dependencies in the data. To address this issue, the LSTM networks have emerged as a successful solution [35]. The LSTM network is a neural network structure designed for processing long sequence data, addressing the issues of gradient explosion or disappearance in traditional RNN [35,57]. In LSTM, internal recurrent loops make it to retain earlier data and establish temporal dependencies between consecutive data points. Its core is the cell state and the “gate” structure. The cell state is a crucial variable that enables the transfer of information from earlier steps throughout the entire network. The process consists of three key steps: forgetting, inputting, and outputting, which are realized by three “gates”. Specifically, the “gate” structure of LSTM comprises the forget gate, the input gate, and the output gate [58–60]. The structure of LSTM is shown in Fig 3. The basic principle is as follows:

1. The input of the forget gate *f*_*t*_, the input gate *i*_*t*_, and the output gate *o*_*t*_ are all taken from the input *x*_*t*_ of the current time step and the output *h*_*t-1*_ of the previous time step, using a sigmoid activation function such that the output is between 0 and 1. The difference between these gates is the weights and bias values of the inputs. The relevant formulas are as follows:

$$\text{sig}moid(x)=\frac{1}{1+e^{-x}}$$ (1)

$$f_{t}=sigmoid(W_{f}\cdot [h_{t-1},x_{t}]+b_{f})$$ (2)

$$i_{t}=sigmoid(W_{i}\cdot [h_{t-1},x_{t}]+b_{i})$$ (3)

$$o_{t}=sigmoid(W_{o}\cdot [h_{t-1},x_{t}]+b_{o})$$ (4)

where *W*_*f*_, *b*_*f*_, *W*_*i*_, *b*_*i*_, *W*_*o*_, and *b*_*o*_ denote the input weights and biases of the forget gate, the input gate, and the output gate, respectively. And *h*_*t-1*_ and *x*_*t*_ denote the output at moment *t*-1 and the input at moment *t*.

2. *c*_*t*_ represents the state of the unit cell. It enables the LSTM model to learn the long-term dependencies well. The formulas are defined as follows:

$$\tanh\text{(}x\text{)}=\frac{e^{x}-e^{-x}}{e^{x}+e^{-x}}$$ (5)

$$c_{t}=f_{t}*c_{t-1}+i_{t}*\tanh\text{(}W_{c}\cdot \text{[}h_{t\text{-1}},x_{t}\text{]+}b_{c}\text{)}$$ (6)

where *f*_*t*_ is the output of the forget gate, *c*_*t-1*_ is the state of the unit cell at the moment *t*-1, it is the output of the input gate, and *W*_*c*_ and *b*_*c*_ are the weights and bias values.

3. *h*_*t*_ represents the final output of the LSTM model at time step *t*. The formulas are defined as follows:

$$h_{t}=\tanh\text{(}c_{t}\text{)}*o_{t}$$ (7)

where *c*_*t*_ is the output of the unit cell at time *t* and *o*_*t*_ is the output of the output gate.

### 3.2. LSTM autoencoder

**Fig 4:**
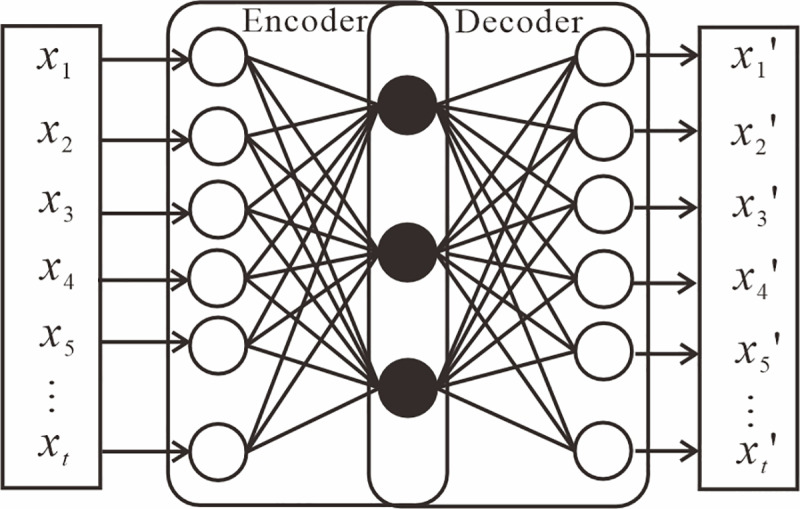
AE model architecture.

**Fig 5:**
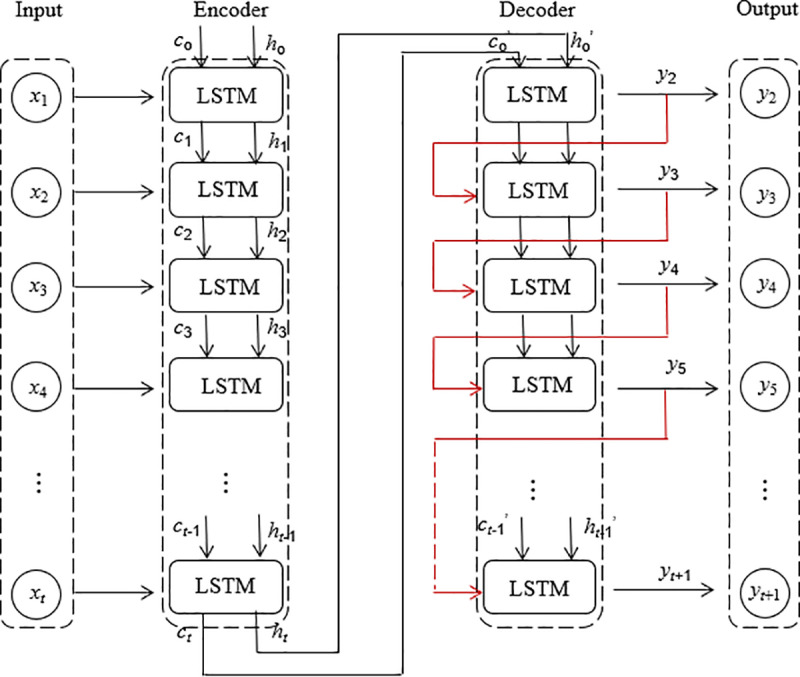
LSTM-AE framework.

Autoencoder (AE) is a widely used unsupervised learning algorithm designed to achieve dimensionality reduction and feature extraction of data by learning an efficient encoding of unlabeled data [62–63]. It consists of an encoder and a decoder. The goal of the encoder is to convert the input data *x* into a low-dimensional representation. The goal of the decoder is to map this low-dimensional representation back to the original state and reconstruct the input data [64–65]. This type of model enhances performance by modifying hidden layers in various ways. When a neural network is deep, the vanishing gradient problem is addressed by stacking hidden layers [66]. The structure of the AE model is shown in Fig 4. LSTM-AE combines the ideas of LSTM and AE, which uses LSTM units to build the encoder and the decoder, thus enabling the encoder and decoder to process time series data or time-dependent data [67–68]. The LSTM-AE framework is shown in Fig 5. The input data is the time series data within a time step *t*. The hidden state *h*_*t*_ and the memory cell state *c*_*t*_ at the last moment of the encoder are obtained after the computation of the LSTM unit, which is passed into the first LSTM of the decoder as initial states for prediction.

### 3.3. Indicators for the assessment

Four evaluation metrics commonly used in regression tasks are used to assess the goodness of the model, and they are defined in Eqs 8–11. The mean square error (*MSE)*, root mean square error (*RMSE*), and mean absolute error (*MAE*) are all used to measure the error between the predicted value and the true value, and their values are all as small as possible. The coefficient of determination (*R*^*2*^) is used to measure the ability of the model to fit the data and its value range is [0, 1]. The closer its value is to 1, the better the model fits. Due to their different calculation methods, each metric is affected differently by outliers. Thus the combined use of these four indicators helps to assess the model more comprehensively.


$$MSE=\frac{1}{m}\sum_{i=1}^{m}{\text{(}y_{i}-{\overset{⌢}{y}}_{i}\text{)}}^{2}$$ (8)

$$RMSE=\sqrt{\frac{1}{m}\sum_{i=1}^{m}{\text{(}y_{i}-{\overset{⌢}{y}}_{i}\text{)}}^{2}}$$ (9)

$$MAE=\frac{1}{m}\sum_{i=1}^{m}\left|\text{(}y_{i}-{\overset{⌢}{y}}_{i}\text{)}\right|$$ (10)

$$R^{2}=1-\frac{\sum_{i=1}^{m}{\text{(}{\overset{⌢}{y}}_{i}-y_{i}\text{)}}^{2}}{\sum_{i=1}^{m}{\text{(}y_{i}-{\bar{y}}_{i}\text{)}}^{2}}$$ (11)

Where $y_{i}$ represents the true value, ${\hat{y}}_{i}$ represents the predicted value, ${\bar{y}}_{i}$ represents the sample average and *m* represents the number of samples.

## 4. Experiments and results

**Fig 6:**
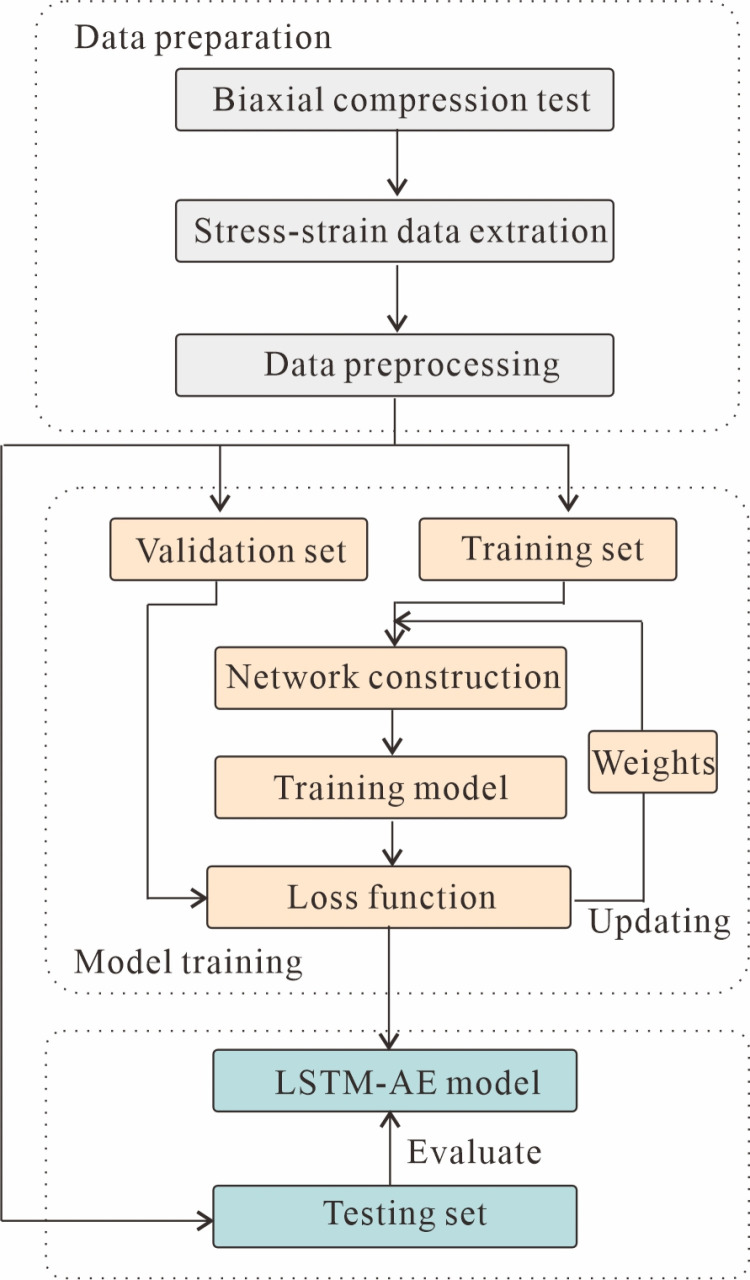
Flowchart of LSTM-AE network in stress-strain curve prediction.

This section displays the prediction results of the LSTM-AE model and its accuracy in predicting stress-strain curves of rock materials through an in-depth examination. Fig 6 illustrates the flowchart for predicting the stress-strain curve. Next, some details of the experiment, the results, and a discussion of the results will be presented.

### 4.1. Data preprocessing and modeling details

In order to make the data collected in section 2.2 fit the inputs to the model, preprocessing of the dataset is required. Therefore, the coefficient of friction (*μ*), confining pressure (*σ*_*3*_), tensile strength (*T*_*b*_), shear strength (*C*_*b*_), different strains (*ε*), and the deviatoric stress (*σ*_1_-*σ*_3_) at the previous moment are taken as inputs and the output is the deviatoric stress at the next moment. To ensure that the model is better trained, the input data needs to be scaled to a specific range, i.e., standardized. Additionally, the data of each curve is transformed appropriately so that it can be used for training and testing the network model [37]. As shown in Fig 6, the time series data is converted to LSTM training data using the sliding window method [69].

**Fig 7:**
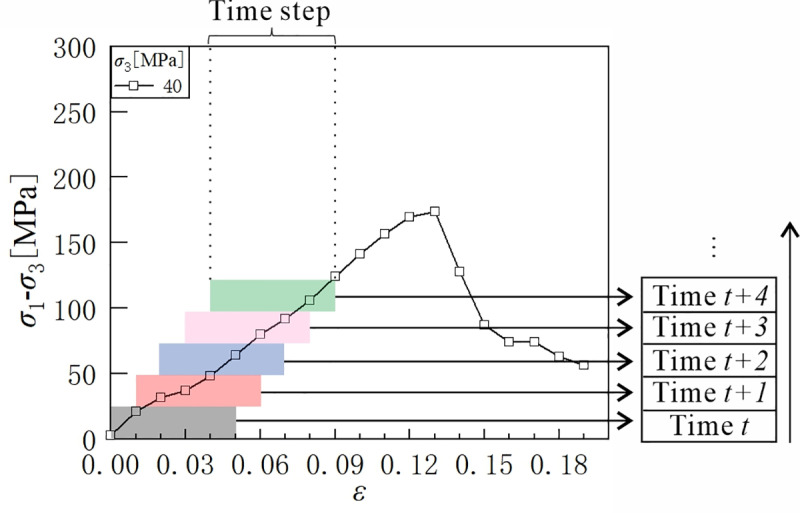
Processing time series data using the sliding window method, where *t* represents the label of the time series segment.

In the model-building process, the prepared data is first divided into training, validation, and testing sets, with the training set comprising 70% of the data, and the validation and testing sets each comprising 15%. Next, the model structure, consisting of an encoder and a decoder, is constructed using LSTM. During training, the model’s performance is evaluated using validation sets, and training is stopped when there is no significant improvement in the model performance after several iterations. Table 3 represents the parameters setting of the LSTM-AE model, which were manually tuned through iterative trials, with the optimal configuration selected based on validation score. Among these, the learning rate and dropout rate are two key hyperparameters. Specifically, the learning rate controls the step size of the parameter update of the model during the training process. Choosing an appropriate learning rate can accelerate the convergence of the model, improve the training efficiency, and enhance the generalization ability of the model. If the learning rate is too large, it may cause the model to fail to converge because the step size of the parameter update is too large, and the optimal solution may be missed; while if the learning rate is too small, although the convergence is stable, the training process is too slow, which may make the model fall into the local optimal solution. The dropout rate, on the other hand, controls the proportion of nodes randomly dropped in the neural network. It reduces the model’s dependence on some specific nodes by randomly dropping some neurons during the training process, thus enhancing the model’s generalization ability. If the dropout rate is set too high, too many neurons are discarded during each training, which may make the training process unstable; while if it is set too low, the model may be overfitted on the training set, resulting in poor generalization ability. Fig 7 illustrates the time series prediction details for a time step. It is important to highlight that the time step discussed here pertains to the time series data, whereas in Table 1, it refers to a parameter associated with the DEM.

**Table 3:** Parameters of the LSTM-AE model.

Parameter	Value
Input size	6
Hidden layers	64
Lstm layers	2
Epochs	100
Batch size	128
Learning rate	1e^-3^
Optimizer	*Adam*
Loss function	*MSE*
Dropout	0.2
Time step	5
Output size	1

### 4.2. Predictive results of the proposed model

**Fig 8:**
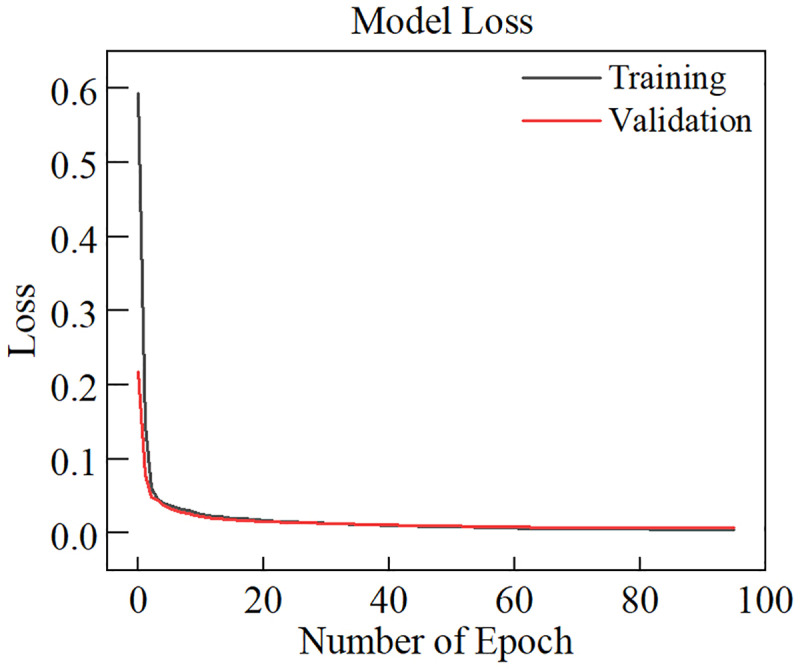
Loss on the training and validation sets in the training process.

**Fig 9:**
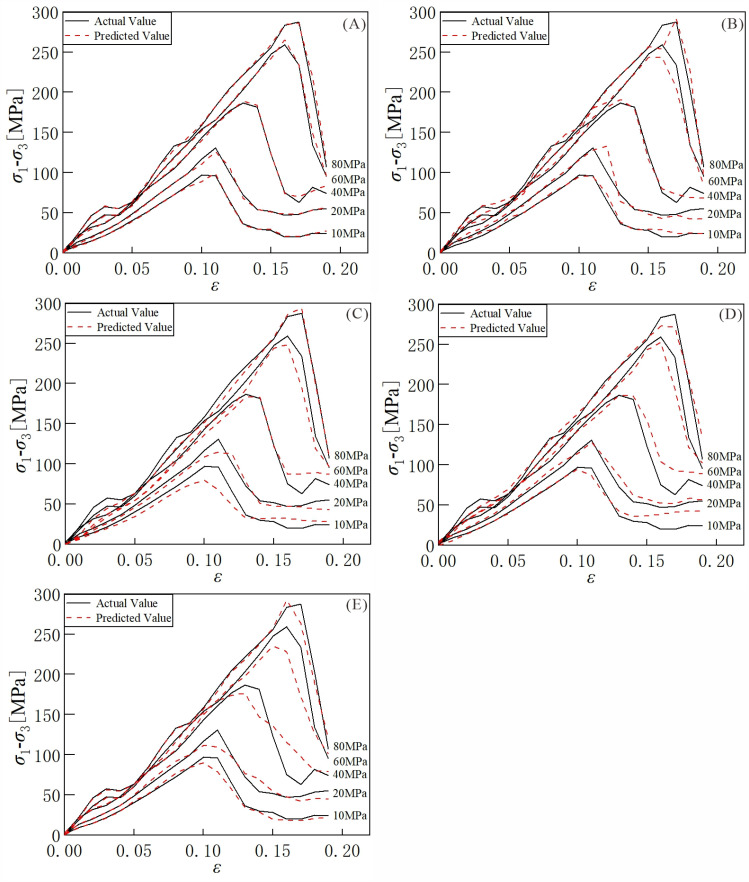
Prediction results of different models on specific samples. (a) LSTM-AE, (b) LSTM, (c) RNN, (d) BPNN, (e) XGBoost.

**Fig 10:**
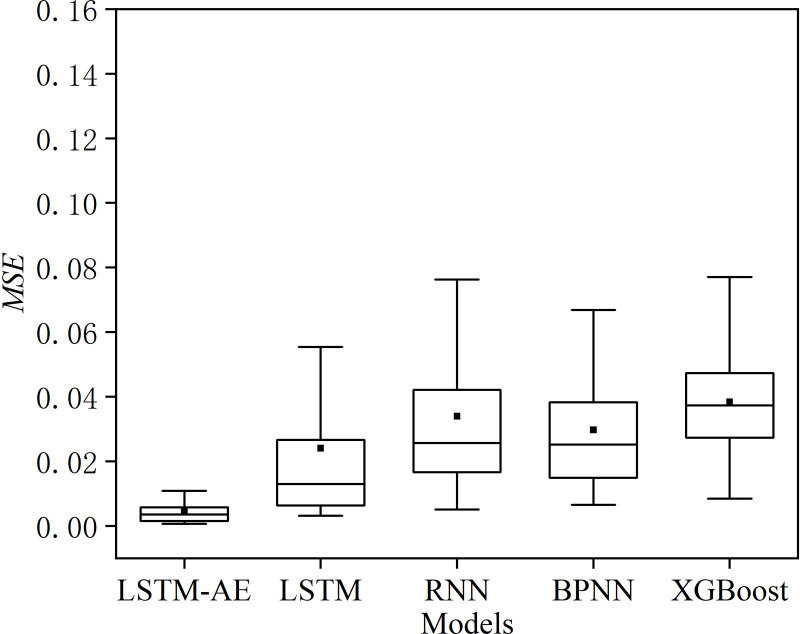
Boxplots for the *MSE* of the different models in the testing set.

Fig 8 shows loss of the model in the training process. As shown in Fig 8, the model’s loss decreases gradually in the initial stage. As the number of training epoch increases, both the training loss and validation loss stabilize at a low level, indicating that the model has converged. Table 4 shows the results of the evaluation metrics for different models. Fig 9 show the prediction results of different models for specific samples. From the table, the LSTM model has lower *MSE*, *RMSE*, *MAE,* and higher *R*^*2*^ compared to the RNN, BPNN, and XGBoost models. However, the LSTM model still requires further enhancement in predicting the stress-strain curves of rock materials. Overall, the improvement of the proposed model is significant. Compared to the LSTM model, the LSTM-AE model reduces the *MSE*, *RMSE*, and *MAE* by 0.0191, 0.08494, and 0.04745, respectively. The *R*^*2*^ improves by 0.01599. Fig 10 illustrates the *MSE* distribution of different models on the testing set. The LSTM-AE model exhibits a lower *MSE* than the other models, with a more concentrated error distribution. These results indicate that the proposed model has a less error between the predicted value and the true value, and it has a stronger prediction ability and data fitting ability. In contrast, the prediction results of the other four models under specific samples show significant deviations.

**Table 4:** The forecasting results in different models.

Metric/Model	LSTM-AE	LSTM	RNN	BPNN	XGBoost
*MSE*	**0.00489**	0.02399	0.03311	0.02953	0.03604
*RMSE*	**0.06993**	0.15487	0.18197	0.17183	0.18983
*MAE*	**0.03993**	0.08738	0.11145	0.09467	0.10036
*R* ^*2*^	**0.99591**	0.97992	0.97086	0.97439	0.96985

### 4.3. The robustness of the LSTM-AE network

**Fig 11:**
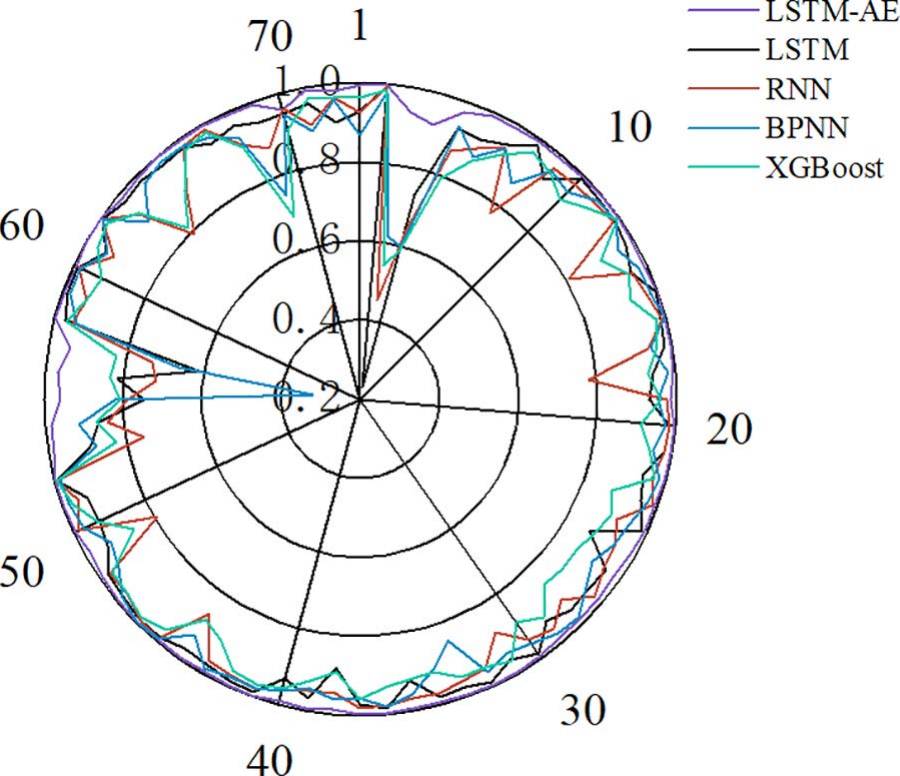
Coefficient of determination of each group of samples for different models on the testing set.

**Fig 12:**
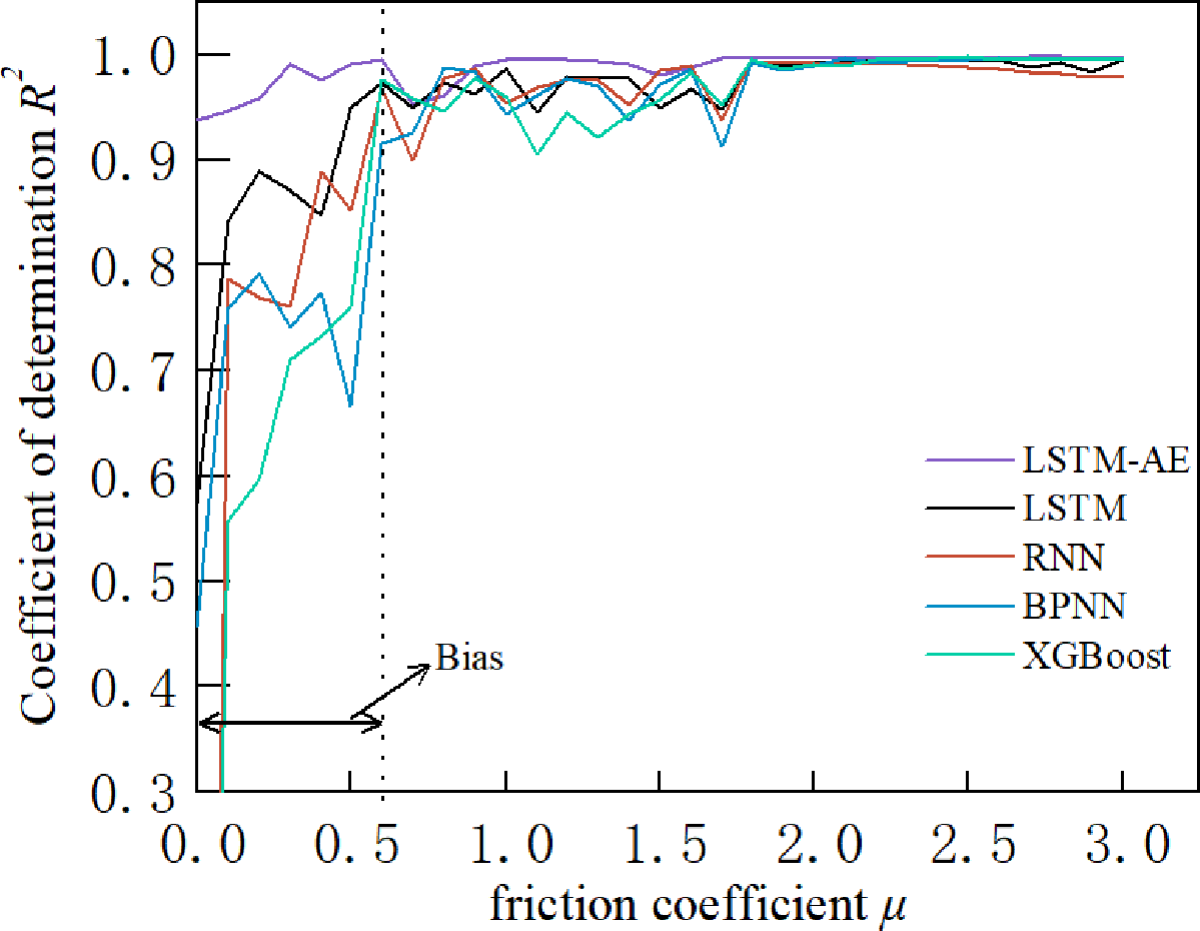
Coefficients of determination for different models at different friction coefficients.

**Fig 13:**
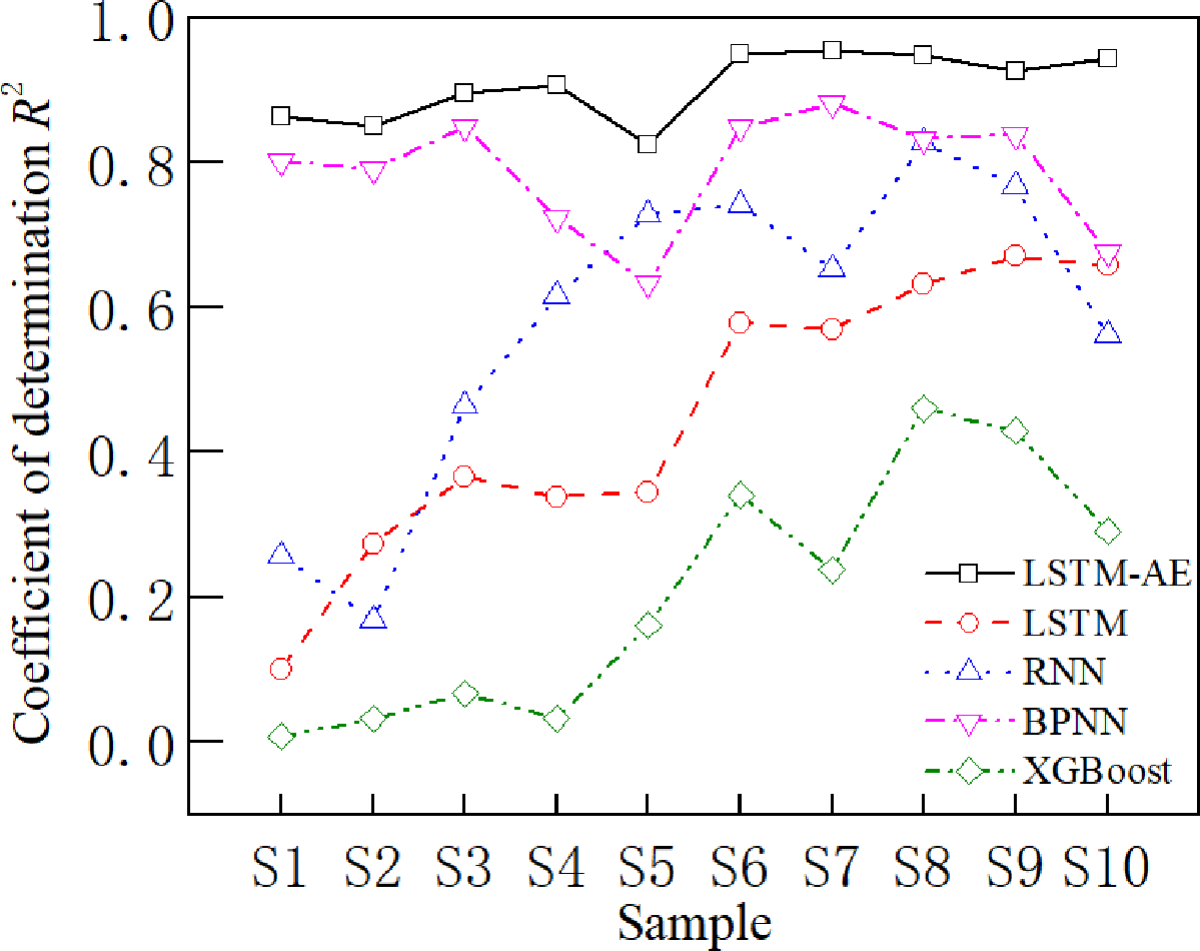
Coefficients of determination of different models for special samples.

In general, some samples affect the prediction accuracy of stress-strain curves in practical applications. Therefore, in this section, the influence of the microscopic parameters of the rock material on the prediction accuracy of the stress-strain curve is analyzed and verifies the robustness of the LSTM-AE model. Samples with coefficients of determination less than 0.8 were first analyzed, and the coefficients of determination for the different models for each group of samples on the testing set are shown in Fig 11. It can be seen that LSTM, RNN, BPNN and XGBoost models have lower prediction accuracy on individual samples. The analysis of the samples with lower prediction accuracies reveals that there is a phenomenon of low confining pressure or low friction coefficient in all of these samples. Thus, in order to investigate the effect of different confining pressures and friction coefficients on the prediction accuracy, the coefficients of determination of the five models were examined for different confining pressures and friction coefficients. Fig 12 shows that the coefficient of determination increases with the friction coefficient on LSTM, RNN, BPNN and XGBoost models. Bias exists at low friction coefficients, and high friction coefficients help improve the accuracy of these four models. However, a similar pattern is not shown in the confining pressure. Fortunately, the LSTM-AE model has high accuracy on all these samples. Thus, to further verify the accuracy of the model, 10 sets of microscopic parameters at low friction coefficients and low confining pressures were designed to generate the stress-strain data. The prediction results of LSTM-AE are shown in Fig 13. It can be seen that the proposed LSTM-AE model still exhibits relatively high robustness at low friction coefficients and low confining pressures.

## 5. Discussion

Currently, various numerical techniques are widely available to simulate the stress-strain behavior of rock materials. However, due to the complexity of rock materials, obtaining a rigorous constitutive model is extremely challenging. With the development of deep learning technology, a data-driven approach for researching constitutive models of rock materials has been provided. In this study, a method for predicting stress-strain curves of rock materials in discrete element numerical simulations based on the LSTM-AE model is proposed. The LSTM-AE model has better prediction accuracy as well as robustness compared to the methods often used for prediction. The prediction bias of traditional machine leanring methods primarily arises from their limited ability to capture temporal dependencies, particularly when addressing complex time-series problems. Additionally, the RNN model is susceptible to the gradient vanishing problem, while the lower weight gradients in LSTM models may cause undertraining in low-stress and low- friction samples, resulting in large prediction biases. By leveraging the properties of the self-encoder, LSTM-AE effectively mitigates the influence of noise and better extracts key features from time-series data, thereby improving prediction accuracy for low-stress and low-friction coefficient samples. To further analyze the bias in these samples, future methods such as Explainable Artificial Intelligence (XAI) and SHapley Additive exPlanations (SHAP) algorithms can be integrated to provide deeper insights into the neural network’s inference process, helping address these issues [70,71].

However, only the prediction of stress-strain curves for rock materials in discrete element numerical simulations is discussed in this study. In the future, this method can predict the stress-strain behavior of more complex DEM experiments, and the effects of more microscopic parameters on the accuracy of stress-strain curve prediction can be explored. The aim is to improve the accuracy and reliability of data-driven methods for stress-strain curve prediction of granular materials. In terms of application, it provide an idea to researchers in the field of DEM numerical simulation. When performing DEM experiments, it is often necessary to determine a set of microscopic parameters by continuously adjusting them to produce the stress-strain curve required for the experiment. However, this process is time-consuming and cumbersome, and a data-driven approach makes it possible to solve this problem. By training a large amount of data to get the prediction model, the stress-strain curve can be obtained quickly. Thus, the accuracy of the prediction is particularly important in this process.

In the future, we aim to explore more efficient techniques to replace or enhance existing stress-strain curve prediction models, thereby improving the accuracy and reliability of data-driven approaches. Specifically, we plan to integrate additional microscopic parameters into the model, apply SHAP for feature extraction, and incorporate advanced methods such as attention mechanisms and transfer learning to further improve prediction accuracy and generalization. Furthermore, we will train, calibrate, and validate the model using indoor experimental data. Our goal is to develop a more accurate, robust, and adaptable prediction model, which will significantly contribute to research on stress-strain curve prediction for rock materials.

## 6. Conclusion

The evaluation of stress-strain curves of rock materials is one of the important indicators for analyzing their mechanical properties. However, it is difficult to describe the stress-strain relationship of rock materials with a single constitutive law. Furthermore, existing deep learning methods cannot guarantee the accuracy of the prediction of the stress-strain curve of rock materials. In this study, with the help of discrete element numerical simulation, a deep learning method based on LSTM-AE for predicting stress-strain curves of rock materials is proposed. By comparing with four models commonly used for prediction tasks, which are LSTM, RNN, BPNN, and XGBoost. The results show that the *MSE*, *RMSE*, *MAE,* and *R*^*2*^ of the LSTM-AE model are 0.00489, 0.06993, 0.03993, and 0.99591, respectively, and the four evaluation indexes are better than the other four models, which verifies the accuracy of the LSTM-AE model. In addition to this, special samples were designed to validate the accuracy of the LSTM-AE model again. The proposed method improves the accuracy and reliability of stress-strain curve prediction. In the future, this method can be extended to 3D discrete element numerical simulations. Additionally, it can be applied to predict the stress-strain behavior of rock materials at different scales.

## Supporting information

S1 File Parameters of biaxial compression tests. (TXT)

S2 File Stress-strain data sets. (TXT)
